# Expression of 1N3R-Tau Isoform Inhibits Cell Proliferation by Inducing S Phase Arrest in N2a Cells

**DOI:** 10.1371/journal.pone.0119865

**Published:** 2015-03-30

**Authors:** Li Li, Zhi-Peng Xu, Gong-Ping Liu, Cheng Xu, Zhi-Hao Wang, Xiao-Guang Li, En-Jie Liu, Juan Zeng, Da-Min Chai, Wen-Long Yao, Jian-Zhi Wang

**Affiliations:** 1 Department of Pathophysiology, Key Laboratory of Education Ministry of China for Neurological Disorders, Tongji Medical College, Huazhong University of Science and Technology, Wuhan, China; 2 Co-innovation Center of Neuroregeneration, Nantong University, Nantong, China; 3 Department of Anesthesiology, Tongji Hospital, Tongji Medical College, Huazhong University of Science and Technology, Wuhan, China; Cleveland Clinic Foundation, UNITED STATES

## Abstract

Tau is a microtubule-associated protein implicated in neurodegenerative tauopathies. Six tau isoforms are generated from a single gene through alternative splicing of exons 2, 3 and 10 in human brain. Differential expression of tau isoforms has been detected in different brain areas, during neurodevelopment and in neurodegenerative disorders. However, the biological significance of different tau isoforms is not clear. Here, we investigated the individual effect of six different isoforms of tau on cell proliferation and the possible mechanisms by transient expression of eGFP-labeled tau isoform plasmid in N2a cells. Our study showed the transfection efficiency was comparable between different isoforms of tau by examining GFP expression. Compared with other isoforms, we found expression of 1N3R-tau significantly inhibited cell proliferation by Cell Counting Kit-8 assay and BrdU incorporation. Flow cytometry analysis further showed expression of 1N3R-tau induced S phase arrest. Compared with the longest isoform of tau, expression of 1N3R-tau induced cyclin E translocation from the nuclei to cytoplasm, while it did not change the level of cell cycle checkpoint proteins. These data indicate that 1N3R-tau inhibits cell proliferation through inducing S phase arrest.

## Introduction

Tau is a microtubule-associated protein, which is mainly expressed in the axon of neuronal cells. The major function of tau is to promote microtubule assembly and stabilization, thus contributing to the integrity of the cytoskeleton and the maintenance of intact axonal transport. Tau dysfunction, such as increased phosphorylation and aggregation, has been correlated with many neurodegenerative diseases, including Alzheimer’s disease (AD) and related dementias [1,2].

In human brain, six tau isoforms are generated from a single gene through alternative splicing of exons 2, 3 and 10. They were 0N3R, 1N3R, 2N3R, 0N4R, 1N4R and 2N4R, according to the numbers of C-terminal microtubule-binding repeats (3 or 4) and the numbers (0, 1, or 2) of N-terminal inserts [3,4].

Previous studies have demonstrated that expression of tau isoforms is differentially regulated during development [5–7]. The fetal brain predominantly expresses the shortest (0N3R tau) isoform, whereas the adult brain expresses all six isoforms. In addition, there is regional and quantitative variation of tau isoforms expression. In adult brain, the proportion of 1N-tau isoforms is higher than 0N-tau isoforms and 2N-tau isoforms, and the levels of 3R-tau and 4R-tau isoforms were approximately equal [8,9]. Meanwhile, the differential distribution of tau isoforms in brain region has been reported recently by several groups [8–12]. Moreover, the composition of tau isoforms was also different in many neurodegenerative diseases. Conrad et al found an up-regulation of 0N4R-tau and a decrease of 2N3R and 1N3R-tau in Alzheimer’s disease [13]. In the brain of myotonic dystrophy type I, Sergeant et al found that the pathological tau proteins mainly consisted of tau isoforms without exon 2 [14]. These evidences imply that different tau isoforms must play specific roles in neurodevelopment and neurodegenerative disorders. However, the exact pathophysiological functions of different tau isoforms have not been illustrated.

Studies suggest that each tau isoform may have different functions due to distinct microtubule-binding ability [15,16], phosphorylation level [17,18] and filament formation [19,20]. In the present study, we aimed to investigate the individual effect of six different isoforms of tau on cell proliferation and possible mechanisms by transient expression of eGFP-labeled tau plasmid in N2a cells.

## Materials and Methods

### Cell culture and transfection

Mouse neuroblastoma 2a (N2a) cells were obtained from Dr Hua-xi Xu (Sanford-Burnham Medical Research Institute, La Jolla, California) [21,22]. The cells were cultured in a 1: 1 mixture of Dulbecco's modified Eagle's medium and OPTI-MEM supplemented with 10% fetal bovine serum (FBS) and grown in a humid atmosphere containing 5% CO_2_ at 37°C. The plasmids pEGFP-tau-0N3R, pEGFP-tau-1N3R, pEGFP-tau-2N3R, pEGFP-tau-0N4R, pEGFP-tau-1N4R and pEGFP-tau-2N4R, encoding alternatively spliced six isoforms of the microtubule-associated protein tau, were generous gifts from Dr. Fei Liu (Jiangsu Key Laboratory of Neuroregeneration). All plasmids were prepared using endotoxin-free plasmid extraction kit. They were sequenced correctly before transfection and the green fluorescent protein (GFP)–tau fusion protein can be examined by western blot.

Cells were seeded at a density of 5 × 10^4^ cells/cm^2^ before transfection. They were transfected with the plasmids using Lipofectamine 2000 (Invitrogen, USA) according to the manufacturer’s instruction. Unless specified otherwise, the analyses were performed at 48 h after transfection. All cell culture experiments were repeated at least three times, and representative pictures were shown for each experiment.

### Cell viability assay

Cell viability was evaluated by colorimetric assay with Cell Counting Kit-8 (CCK8, Dojindo, Kumamoto, Japan) in 96-well plates. Briefly, after 48 h of transfection, the CCK8 reagent (10μl/well) was added to each well and cells were incubated for 1h at 37°C. The absorbance at 450 nm was measured using Synergy 2 multi-mode microplate reader (BioTek, Vermont, USA).

### Cell proliferation by BrdU incorporation

BrdU can be incorporated into the newly synthesized DNA of replicating cells, which is commonly used to assess cell proliferation. At 48 h after transfection, cells were incubated with 10 μM BrdU for 2h. Double immunofluorescent staining was performed with separate antibody incubation. Briefly, cells were fixed with 4% paraformaldehyde. After rinsed with PBS, cells were treated with 2N HCl for 30 min. Nonspecific binding was blocked in 5% bovine serum albumin (BSA) for 1h at room temperature. Cells were respectively incubated with mouse anti-BrdU (1:200, Millipore, MAB3222) and rabbit anti-GFP (1:1000, Abcam, ab290) overnight at 4°C one after another. Then followed by three times washes in PBS, they were incubated with Alexa Fluor 546 Donkey Anti-Mouse IgG (1:1000, Invitrogen) and Alexa Fluor 488 Donkey Anti-Rabbit IgG (1:1000, Invitrogen) for 1h at room temperature. Immunofluorescence was observed using a confocal microscope (Carl Zeiss, Jena, Germany) and images were acquired using ZEN 2009 software (Carl Zeiss, Jena, Germany). The cell proliferation was evaluated by counting BrdU incorporation in eGFP expressing cells.

### Cell-cycle analysis

At 48 h after transfection, cell-cycle distribution was analyzed by flow cytometry (FCM). Cells were fixed with 70% ethanol and stained with 50 μg/ml propidium iodide and 20μg/ml RNase at 37°C for 30 min. Stained cells (1 × 10^4^) were quantified to determine the distribution of different cell cycle phases using Multicycle AV software (FACSAria, BD Biosciences, CA, USA).

### Western blot

Western blotting was performed as reported previously [23]. At 48 h after transfection, the cells were harvested and washed with ice-cold PBS. Total proteins were extracted with RIPA lysis buffer (Beyotime, China). Cytoplasm and nuclear proteins were extracted using the NE-PER Nuclear and Cytoplasmic Extraction Reagent Kit (Pierce, Rockford, IL, USA). The protein concentration was determined using the BCA Protein Assay Kit (Pierce, Rockford, IL, USA). Equal amounts of extracts (40 μg) were separated by electrophoresis in a 10% sodium dodecylsulfate-polyacrylamide gels (SDS-PAGE) and transferred to nitrocellulose filter membranes. After blocking, membranes were incubated with primary antibodies against cyclin A (1:500, Santa Cruz, sc-751), cyclin B1 (1:500, Santa Cruz, sc-752), cyclin D1 (1:500, Santa Cruz, sc-246), cyclin E (1:500, Santa Cruz, sc-481), Cdk2 (1:1000, Millipore, 07–631), Cdk4 (1:1000, Abcam, ab6315), GFP (1:1000, Abcam, ab290), Lamin B1 as a nuclear loading control (1:1000, Abcam, ab16048) or DM1A as a reference gene (α-tubulin, 1:1000, Sigma, T9026) overnight at 4°C, the membranes were then incubated with IRDye 800CW anti-mouse or anti-rabbit IgG (1:10000, LI-COR biosciences) for 1h at room temperature. The immunoreactive bands were scanned and visualized by using the Odyssey Infrared Imaging System (LI-COR biosciences, Lincoln, NE, USA).

### Statistical analysis

The data were expressed as mean ± standard deviation of at least three independent experiments. Statistical analysis of the results was performed using ANOVA with SPSS 19.0 software (IBM, Armonk, New York, USA). *P* <0.05 were considered statistically significant.

## Results

### 1N3R-tau inhibits cell viability by CCK8 assays

**Fig 1:**
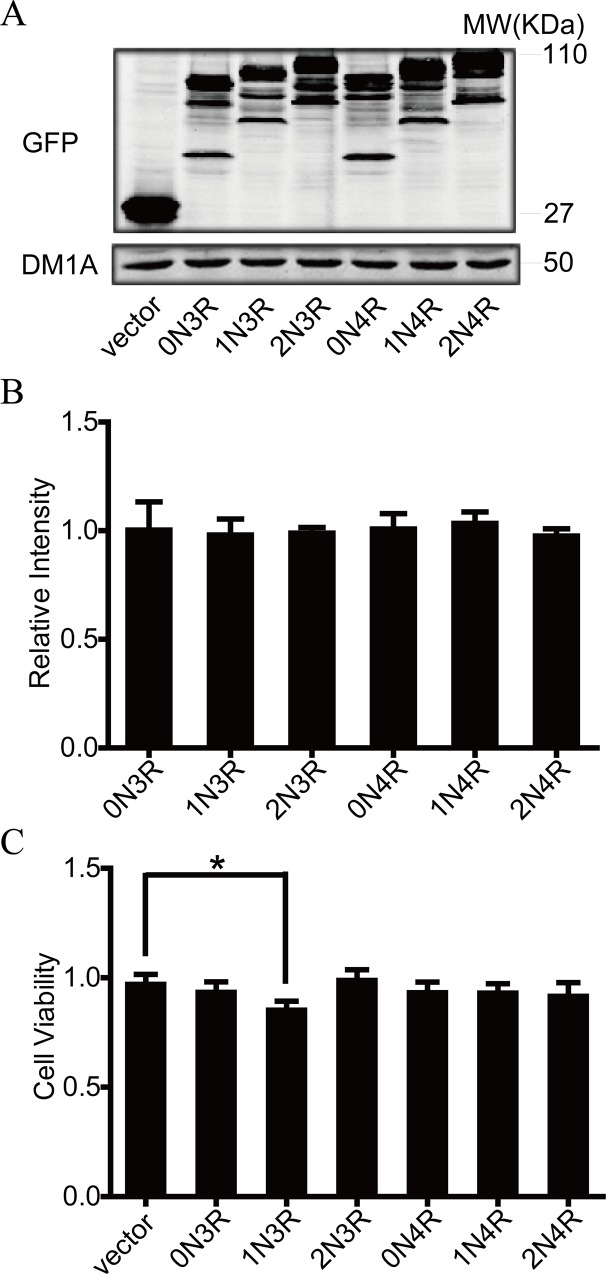
1N3R-tau inhibits cell viability measured by CCK8. **(A)** N2a cells were transfected transiently with six tau isoforms and empty vector for 48 h, then total protein were extracted and eGFP were detected by western blotting. DM1A (α-tubulin) as a reference gene. **(B)** Quantitative analysis of (A). There were no significant differences among tau isoforms. **(C)** CCK8 analysis of cell viability after 48 h transfection with six tau isoforms. All values are standardized with vector. *P < 0.05, compared with vector.

To determine the transfection efficiency, we examined the GFP expression under fluorescent microscopy at 12 h, 24 h, 48 h of transfection. Moreover, the GFP expression was quantified by western blot at 48h of transfection. Our results showed the GFP-tau fusion protein can be detected by western blot. We found many bands in cells trasfected with tau isoforms. The GFP-tau fusion protein was detected at the molecular weight of about 100 kD. Quantitative analysis showed the level of GFP-tau fusion protein was comparable between different tau isoforms (Fig. 1A, B). To explore the effect of different tau isoforms on cell proliferation, we measured cell viability after transfection with different tau isoforms by using CCK8 kit. We observed that transient expression of 1N3R-tau decreased the cell viability when compared with the vector-transfected control cells, while expression of other isoforms of tau did not significantly change the cell viability (Fig. 1C). To exclude the possibility that transfection of 1N3R-tau induced cell death/apoptosis, we did not observe the increase of cellular necrosis in cell culture medium, and nuclear chromatin condensation and fragmentation with Hoechest staining.

### 1N3R-tau inhibits cell proliferation measured by BrdU incorporation

**Fig 2:**
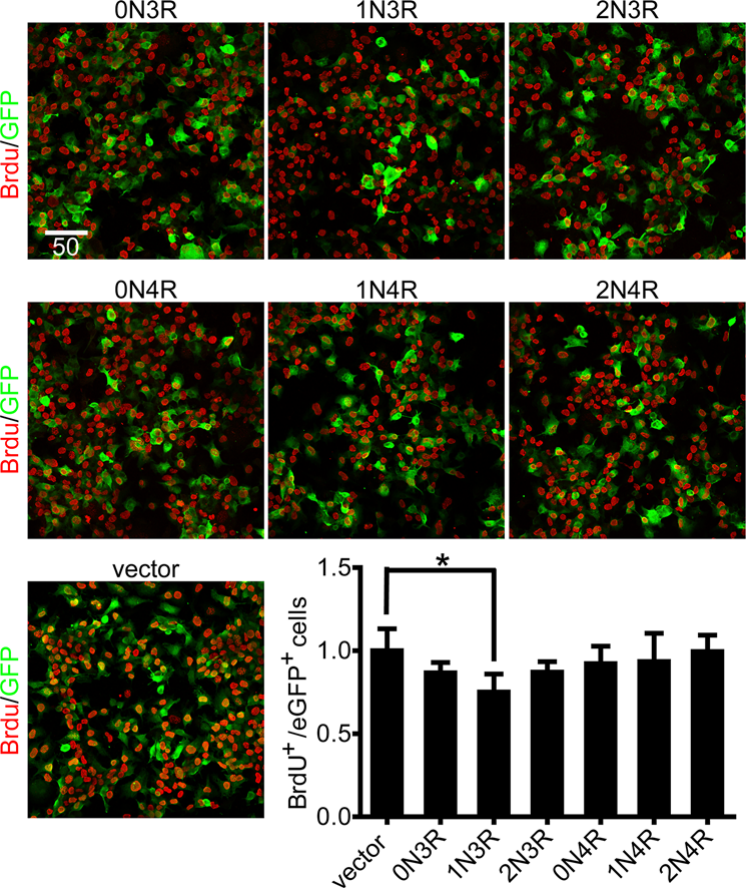
1N3R-tau inhibits cell proliferation measured by BrdU incorporation. Fluorescent micrographs of eGFP and BrdU expression with six tau isoforms in N2a cells after 48 h transfection. GFP (Green), BrdU (Red). Quantification of BrdU-positive cells in eGFP expressing N2a cells with six tau isoforms after 48 h transfection. Scale bar = 50 μm. All values are standardized with vector. *P < 0.05, compared with vector.

N2a cells were transiently transfected with different isoforms of tau or empty vector. BrdU incorporation was applied to evaluate the DNA synthesis and cell proliferation. Cell proliferation was quantified by the percentage of BrdU-positive cells in eGFP expressing cells. Quantitative analysis was performed by counting a total of 300 cells, for each experiment, randomly observed in 6 microscopic fields from three different experiments. Compared with cells transfected with vector, the percentage of BrdU-positive cells in eGFP expressing cells was significantly decreased in cells transfected with 1N3R-tau, while it was not significantly changed in cells transfected with other isoforms of tau (Fig. 2).

### 1N3R-tau induces S phase arrest in HEK293 cells

To assess the effect of different tau isoforms on cell cycle, we first used N2a cells. Since the transfection efficiency of N2a cells was about 50–60%, we have to measure cell cycle in eGFP expressing N2a cells. However, simultaneous detection of eGFP and DNA content using PI by flow cytometry assay was difficult because of the unique nature of these two fluorogenic reagents [24]. We did not see obvious S phase arrest by flow cytometry in N2a cells, because the transfection efficiency was much lower in N2a cells than in HEK293 cells.

**Fig 3:**
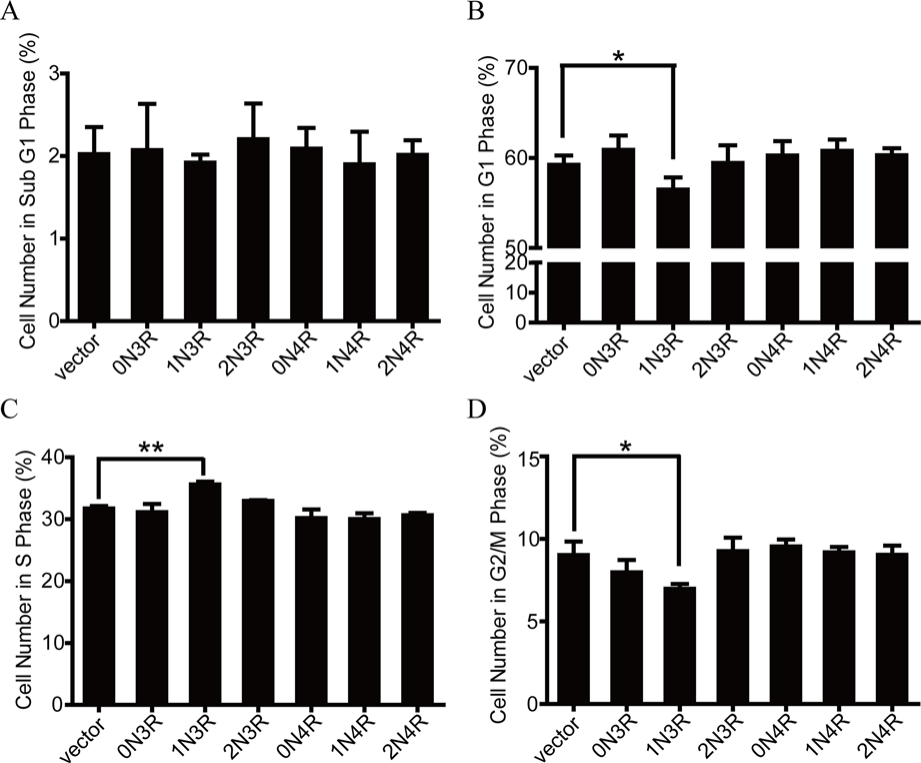
1N3R-tau induces S phase arrest in HEK293 cells. **(A)** The distribution of HEK293 with six tau isoforms in sub G1 phase. **(B)** The distribution of HEK293 with six tau isoforms in G1 phase. *P < 0.05, compared with vector. **(C)** The distribution of HEK293 with six tau isoforms in S phase. **P < 0.01, compared with vector. **(D)** The distribution of HEK293 with six tau isoforms in G2/M phase. *P < 0.05, compared with vector.

Then, we used HEK293 cells in which the transfection rate was about 80–90%. The cells were harvested for flow cytometric analysis at 48 h after transfection of tau plasmids. The results showed there was no significant difference in sub G1 phase among different tau isoforms (Fig. 3A), suggesting that expression of tau isoforms does not induce cell death. Compared with the vector transfected cells, expression of tau 1N3R significantly increased the S phase cell number with a reduced cell number in G1 and G2/M phase, while no significant changes were observed when expression of other tau isoforms (Fig. 3B-D). These data suggest that expression of tau 1N3R induces S phase arrest of the cells.

### 1N3R-tau does not change the level of cell cycle checkpoint protein

**Fig 4:**
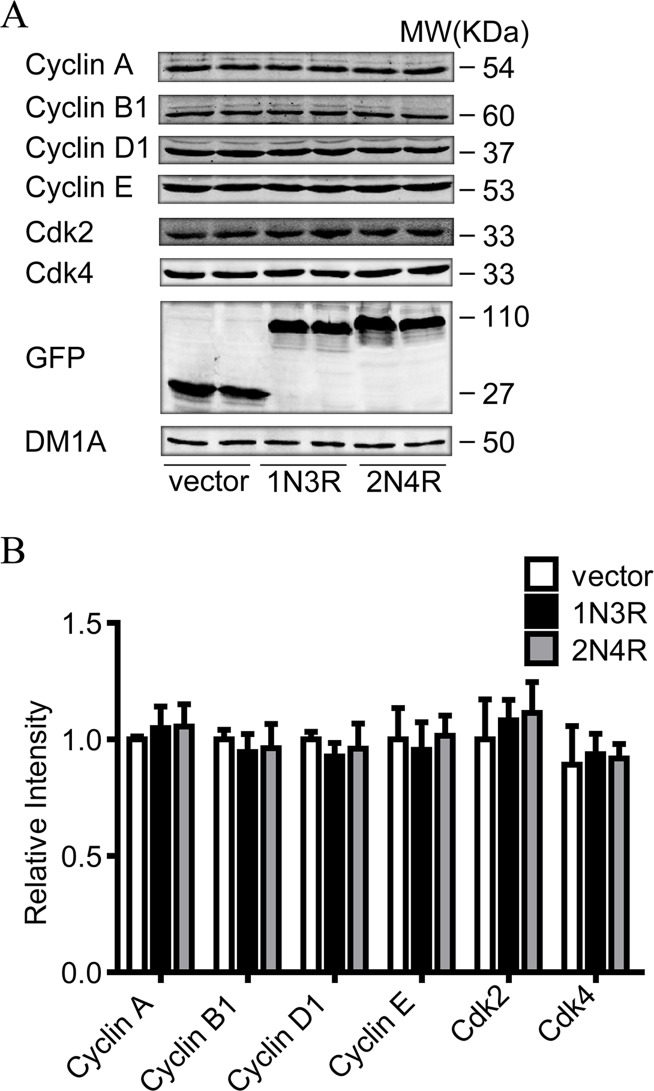
1N3R tau does not affect the expressions of cell cycle checkpoint protein. **(A)** N2a cells were transfected transiently with full length tau (2N4R), 1N3R tau and empty vector for 48 h, and then cyclin A, cyclin B1, cyclin D1, cyclin E, Cdk2, Cdk4 and eGFP were detected by western blotting. DM1A (α-tubulin) as a reference gene. **(B)** Quantitative analysis of (A). All values are standardized with vector. There were no significant differences among three groups.

To investigate the possible mechanism underlying the inhibition of S phase arrest by 1N3R-tau, we examined the expression level of the related cell cycle checkpoint proteins, including cyclin A, cyclin B1, cyclin D1, cyclin E, cyclin-dependent kinase 2 (Cdk2), and Cdk4. N2a cells were transiently transfected with full length tau (2N4R), 1N3R-tau or empty vector. At 48 h after the transfection, the total protein was extracted for western blotting analysis. We found there was no difference in the level of GFP-tau protein between different transfected cells. The results showed that levels of cyclin A, cyclin B1, cyclin D1, cyclin E, Cdk2, and Cdk4 were not changed after expression of 1N3R-tau when compared with expression of vector or 2N4R (Fig. 4).

### 1N3R-tau induces translocation of cyclin E from nucleus to cytoplasm

**Fig 5:**
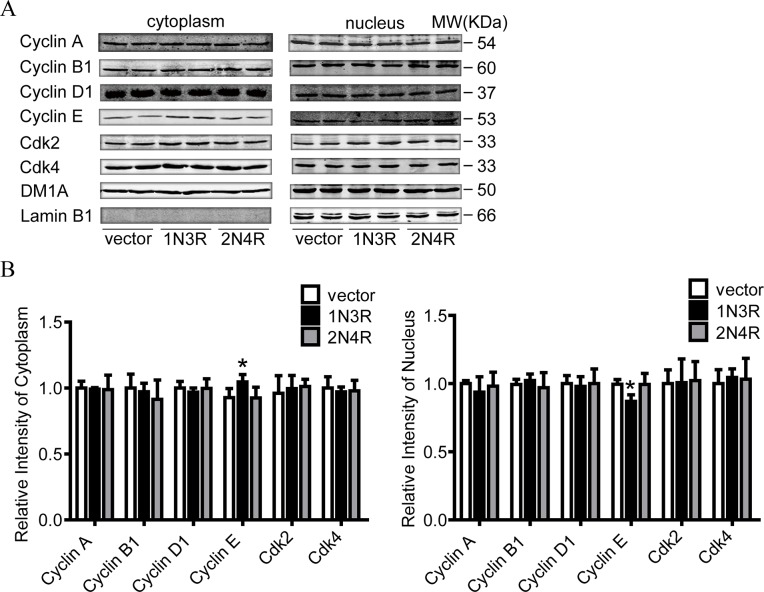
1N3R tau induces translocation of cyclin E from nucleus to cytoplasm. **(A)** N2a cells were transfected transiently with full length tau (2N4R), 1N3R tau and empty vector for 48 h, then cytoplasm and nuclear proteins were extracted and cyclin A, cyclin B1, cyclin D1, cyclin E, Cdk2, Cdk4 were detected by western blotting. DM1A and Lamin B1 represented loading and nuclear reference respectively. **(B)** Quantitative analysis of (A). All values are standardized with vector. *P < 0.05, compared with vector.

Cyclins and their partner cyclin-dependent kinases (Cdks) have been most strongly implicated in controlling entry into and progress through DNA replication. Nuclear localization is crucial to the function of both cyclin A/Cdk2 and cyclin E/Cdk2. Thus, we examined the cytoplasmic and nuclear expression of cyclin A, cyclin B1, cyclin D1, cyclin E, Cdk2, Cdk4. Compared with cells transfected with empty vector or full length tau (2N4R), the cytoplasmic level of cyclin E was increased, while the nuclear evel of cyclin E was decreased in the cells transfected with 1N3R-tau (Fig. 5).

## Discussion

Microtubule-associated protein tau not only regulates the cytoskeleton, but also plays a fundamental role in cell proliferation [25–27], differentiation [5,6], maturation [28] and viability [2,29]. In the central nervous system, there are at least six isoforms of tau with different N-terminal inserts and microtubule-binding domains. Although their primary sequences are very similar, different tau isoforms must have different functions based on their characteristic amino acid sequences and the development-associated expression. Current studies about tau isoforms have been mainly focused on the C-terminal repeat domain (3R-tau and 4R-tau) or the absence and presence of exon in N-terminal (0N-tau, 1N-tau and 2N-tau). In this study, we expressed respectively six tau isoforms into N2a cells to investigate the individual function of each tau isoform. By using CCK8 and BrdU incorporation we found that different from other five tau isoforms, expression of 1N3R-tau suppressed proliferation in N2a cells.

Previous studies also showed that tau isoforms were related with cell proliferation. For instance, expression of human full length tau (2N4R) tau on a murine tau null background mouse model could suppress cell proliferation and promote neuron differentiation in the hippocampus [25]. Expression of human 2N4R tau or the truncated 4R fragment (151–391 amino acids) in SH-SY5Y neuroblastoma cells reduced cell proliferation measured by alamar blue fluorescence analysis [26,27]. Expression of N-terminal 26–230 amino acids of 2N4R tau reduced proliferation of progenitor cells in the adult dentate gyrus [30]. However, it was reported that expression of 2N4R tau did not affect cell proliferation in HEK293 [31] and microglial cells [32]. Similarly, our study showed that expression of 2N4R tau in N2a cells did not significantly affect cell proliferation. The discrepancy may be due to the distinct experimental systems, such as different cells, in vitro or in vivo, employed in the studies.

Cell proliferation is controlled by cell cycle. Studies suggest that reactivation of the cell cycle machinery is involved in tau pathologies [33–35]. We speculate that expression of 1N3R-tau may suppress cell proliferation through regulating cell cycle. To test this, we measured cell cycle by flow cytometry in HEK293 cells after transfection of different tau isoforms. Compared with the cells expressing 2N4R tau or the vector, expression of 1N3R-tau significantly decreased the percentage of G0/G1 and G2/M phase cells with an increased percentage of S phase cells, suggesting that 1N3R-tau can induce S phase arrest.

Different isoforms of tau showed different characteristics of phosphorylation level [17,18], microtubule-binding ability [15,16], and filament formation [19,20]. These differences are largely explained by the presence or absence of exon 10, with some contributions from exons 2 and 3. However, some studies also suggest that 1N3R-tau may have different functions from other tau isoforms. For instance, 1N tau, neither 0N nor 2N, is predominantly expressed in the adult nervous system and the cultured neural cells [8,9]. Compared with 0N and 2N tau isoforms, 1N tau isoforms in the projection domain have the highest negative charge density, which may reduce their overall adhesion due to van der Waals, H-bonding and/or hydrophobic interactions [16]. Before and after phosphorylation by glycogen synthase kinase-3β (GSK-3β), the most significant change in microtubule affinity occurred in 1N3R-tau isoform [17]. Compared with other tau isoforms, expression of 1N3R-tau forms more filamentous protein after 150 μM arachidonic acid treatment [19]. In consistent with these results, our study found that 1N3R-tau isoform has distinct effect on cell proliferation to other isoforms. By comparing with other tau isoforms, we found the inhibition effect of 1N3R tau isoform is not due to the absence of exon 10, since the 1N4R tau did not inhibit cell proliferation. Also the absence of exon 3 is not responsible of this effect. All tau isoforms were expressed as different phosphorylation condition in cells. Although we did not find the detailed mechanism about the different function of 1N3R in this study, it suggested that the specific spatial structure of 1N3R tau isoform itself contributed to this effect, in the presence of exon 2 combined with absence of exon 3 and 10.

Cyclins and cyclin-dependent kinases (Cdks) are the main components that control the progression of cell cycle. A previous study showed that cyclins and Cdks could directly interact with tau protein [36]. To explore the mechanisms underlying the S phase arrest by 1N3R-tau, we examined the protein levels of cyclins and cyclin-dependent kinases by western blotting using a panel of specific antibodies. We found that expression of 1N3R-tau induced a significant translocation of cyclin E from nuclear fraction into the cytoplasmic fraction, but it did not affect the total protein level when compared with 2N4R tau. The cyclin shuttle between nucleus and cytoplasm is a key regulator of DNA synthesis [37]. Cyclin E had been shown to modify toxicity in fly models induced by both mutant and wild-type tau [38]. In the fly retina expressed with 2N4R tau, a functional genetic screen demonstrated the loss of function in cyclin E [39]. Among various cyclins and Cdks, we found in the present study that only cyclin E was translocated from nuclei into the cytoplasm by expression of 1N3R-tau in N2a cells, which provide new evidence supporting a critical role of cyclin E subcellular localization in 1N3R-tau-induced cell proliferation.

During neurodevelopment, 1N3R-tau is the second tau isoform expressed followed by fetal tau (0N3R). It can be detected in the hippocampal cells at postnatal 4 days, which is a critical period of transition from neural cell proliferation to differentiation [40]. Therefore, our results suggest that the inhibition effect of 1N3R-tau on cell proliferation may play an important role in neurodevelopment.

The limitation is that we did not construct stable cell lines with different tau isoforms. It could be better for understanding the comprehensive function of tau isoforms on cell proliferation. In our study, N2a cells were transiently transfected with different vectors because there was not eukaryocytic resistance in the vectors.

In a summary, by respective transfection of each tau isoform, we have found that expression of 1N3R-tau inhibits cell proliferation with the mechanisms involving S phase arrest in N2a cells. Our finding reveals the intrinsic link between specific tau isoforms and cell proliferation. It will be helpful to further study the differential expression of tau isoforms during neurodevelopment.
